# Improvement of sand-washing performance and internal flow field analysis of a novel downhole sand removal device

**DOI:** 10.1038/s41598-024-64751-9

**Published:** 2024-07-05

**Authors:** Zhiliang Wang, Zhigang Fang, Zhensong Wang, Manlai Zhang, Ruiquan Liao

**Affiliations:** 1 School of Petroleum Engineering, Yangtze University, Wuhan, 430100 Hubei China; 2 Lift Test Base of China National Petroleum Corporation, Hami, 839009 Xinjiang China; 3 Research and Development Center, Tuha Oilfield Company, CNPC, Hami, 839009 Xinjiang China; 4 School of Mechanical Engineering, Yangtze University, Jingzhou, 434023 Hubei China

**Keywords:** Novel sand removal device, Sand washing nozzle, Complete sand washing depth, Sand washing performance, Computational fluid dynamics, Engineering, Chemical engineering, Energy infrastructure, Mechanical engineering

## Abstract

With the progression of many shale gas wells in the Sichuan-Chongqing region of China into the middle and late stages of exploitation, the problem of sand production in these wells is a primary factor influencing production. Failure to implement measures to remove sand from the gas wells will lead to a sharp decline in production after a certain period of exploitation. Moreover, As the amount of sand produced in the well increases, the production layer will be potentially buried by sand. To boost the production of shale gas wells in the Sichuan-Chongqing region and improve production efficiency, a novel downhole jet sand-washing device has been developed. Upon analyzing the device's overall structure, it is revealed that the device adopts a structural design integrating a jet pump with an efficient sand- washing nozzle, providing dual capabilities for jet sand- washing and sand conveying via negative pressure. To enhance the sand- washing and unblocking performance of the device, various sand- washing fluids and the structures of different sand- washing nozzles are compared for selection, aiming to elevate the device's sand- washing and unblocking performance from a macro perspective. Subsequently, drawing on simulation and internal flow field analysis of the device's sand- washing and unblocking process through CFD and the control variable method, it is ultimately found that the length diameter ratio of the cylindrical segment of the nozzle outlet, the outlet diameter, and the contraction angle of the nozzle greatly influence the device's sand- washing and unblocking performance. And the optimum ranges for the length-diameter ratio of the cylindrical segment of the nozzle outlet, the outlet diameter, the contraction angle of the nozzle, and the inlet diameter are 2 to 4, 6 mm to 10 mm, 12° to 16°, and 18 mm and 22 mm, respectively. The findings of the research not only provide new insights into existing sand removal processes but also offer a novel structure for current downhole sand removal devices and a specific range for the optimal size of the nozzle.

## Introduction

As the domestic shale gas extraction technology^1^ continues to develop, China has gradually realized the development leap from conventional gas reservoirs^2^ to complex gas reservoirs^3^ (such as tight and low-permeability gas reservoirs, volcanic gas reservoirs, and high-sulfur carbonate gas reservoirs). According to data from the Ministry of Natural Resources, China’s shale gas resource reserves are estimated to be about 403 billion cubic meters, which are mainly in the Sichuan-Chongqing region^4,5^. Shale gas is a typical unconventional oil and gas resource with high extraction difficulty and development cost^6^. With the continuous exploitation of shale gas fields in the Sichuan-Chongqing region, some of the original old wells have developed different degrees of sand production. Additionally, new wells have been put into production as early as possible, making fractures formed during the fracturing process start discharging fluids before they completely close, resulting in sand production in the initial stage of well operation^7,8^. After a certain period of exploitation, both old and new wells may experience major safety accidents^8^ such as sand burying production layers, sand blocking pumps, rod and pipe breakage, and gas well shutdown. Therefore, improving sand removal capabilities of tools in shale gas wells (bringing settled sand from the wellbore to the ground^9^), reducing production costs during gas production, and reducing the occurrence of underground safety accidents remain research priorities and urgent issues^10^ for gas extraction projects in the Sichuan-Chongqing region.

Currently, underground sand removal technologies at home and abroad mainly include mechanical sand dredging^11^ and hydraulic flushing^12,13^. The former uses a sand dredging tool put into the well to scoop up the settled sand through mechanical reciprocating movement, and the sand that is dredged up is stored in the sand dredging tool (once the sand volume reaches a certain level, the tool needs to be taken out). The latter utilizes high-pressure water jet technology^14^ (after passing through the nozzle, high-pressure fluid forms a high-speed water jet, which utilizes its high kinetic energy to lash against objects^15^) to first disperse the settled sand in the well, and then uses flushing fluid to carry the sand particles to the ground, thus completing the sand removal inside the well. Taking into consideration complex well conditions, sand removal efficiency, technical maturity, scope of application, and production costs, hydraulic flushing is superior to mechanical sand dredging in terms of overall performance. From the perspective of promotion, hydraulic flushing is also a preferred choice for most of the severely sand-producing wells in China, which further proves that hydraulic flushing technology is better than mechanical sand dredging technology.

Concerning hydraulic flushing technology, the flushing methods mainly include obverse flushing^16^ (flushing fluid enters through the oil tube and exits through the annulus between the oil tube and casing), reverse flushing (flushing fluid enters through the annulus between the oil tube and casing and exits through the oil tube), and combined obverse-reverse flushing^17^ (adopting obverse flushing to suspend sand particles, and quickly changing to reverse flushing after the flushing fluid goes through a converter, ultimately carrying the sand particles to the ground^8^). However, for both the flushing method and the flushing device adopted, the nozzle is a key component. The performance of the nozzle directly affects the efficiency of sand removal in the well. Therefore, internal flow field analysis, structural size design, and application effect research on nozzles^18^ are fundamental to ensuring efficient sand removal in wells.

Scholars both domestically and internationally have conducted extensive analysis and research on nozzles. For the analysis of the internal flow field in nozzles, Kim and Park^19^ utilized the Reynolds Stress Model (RSM) to investigate the turbulent flows at the inlets of non-circular (square and triangular) nozzles. Their findings revealed that secondary flows in square and triangular nozzles were stronger compared to those in circular nozzles, and the evolution of these secondary flows is intricately linked with the axial vortical motions in the nozzle. Jiang et al.^20^ and his team employed an indoor visualization experimental setup and Particle Image Velocimetry (PIV) technology to study the internal flow field in nozzles, focusing on the effects of variables like contraction angles on nozzle jetting performance. Their research, integrating experimental observations and theoretical analyses, revealed the presence of vortical flows at the nozzle throat and the entry of the contraction segment. They ascertained that the throat length and contraction angle have optimal values that significantly affect the extent of the high-velocity core region. Horisawa et al.^21^ utilized the Direct Simulation Monte Carlo (DSMC) method for numerical simulation analyses of the interiors of micro-sized single nozzles and micro nozzle arrays. Their findings indicated that smaller nozzles exhibit lower Reynolds numbers, resulting in increased fluid viscosity losses and a resultant decline in nozzle efficiency. Jiang et al.^22^ analyzed the boundary layer flow inside conical nozzles through numerical simulations. Their findings indicate that at lower inlet speeds (approximately 1 m/s), the boundary layer flow on the inner walls of the nozzle is mainly laminar, with the bulk of the energy loss in high-velocity fluids (flow resistance) occurring in the throat area. The length of the throat is strongly linked to the core area and length of the flow. Further, Jiang et al.^23^ and his team applied the Large Eddy Simulation (LES) model and Computational Fluid Dynamics (CFD) to study the flow characteristics of fluids near the nozzle walls. They found that the highest flow speeds in the nozzle are located near the walls in the contraction section, and the shear stress and friction resistance coefficients near the walls in the straight section show considerable periodic variation. Despite widespread research into nozzle internal flow fields, the factors impacting the length of the flow core and their influence patterns have been largely unexplored. Since nozzle performance is directly related to the core flow length and the extent of flow velocity reduction at the nozzle's outlet, it is crucial to investigate the factors affecting core flow length to offer theoretical support for designing nozzles that achieve higher jetting performance and reduced energy loss.

Chen et al.^24^, through their research into the streamline structure within nozzles and referencing airfoil curve designs, engineered a novel type of streamlined nozzle. Their comparative analysis between this new nozzle and traditional conical nozzles revealed that the new design resulted in lower energy losses and reduced jet spread. Ouyang^25^ crafted an innovative nozzle configuration that includes a transitional section. His exploration, utilizing numerical modeling and indoor testing, of how the length of the transitional section and the bevel angle influence nozzle efficiency showed that the optimal configuration—a 45° bevel and a 2 mm transitional section—yielded the least energy loss at the nozzle's entrance and achieved a 17% increase in exit velocity over conventional nozzles. Zhang et al.^26^ conducted a study on the performance of Helmholtz nozzles (self-oscillating nozzles). Unlike conventional nozzles, a distinctive feature of the Helmholtz nozzle is its internal fluid oscillation chamber. Through numerical simulations and experiments, they identified that several critical dimensions of the Helmholtz nozzle (the inner diameter and length of the oscillation chamber, and the nozzle exit diameter) significantly affect the pulsed jet at the nozzle exit. Furthermore, they noted that this pulsed jet is not a true "pulse jet" as it comprises a sequence of high-speed continuous "water bullets" and "lower water bullets"^27^. In their research, Yang et al.^28^ compared the cavitation jet characteristics of angular nozzles with those of accordion nozzles. They observed that the highest fluid velocities are present in both nozzle designs' cylindrical segments. The reduced diameter of this segment creates local negative pressure as the high-velocity fluid passes through, facilitating the generation of cavitation bubbles. Furthermore, they noted that angular nozzles outperform accordion nozzles in cavitation efficiency under certain operating parameters. The research by Liao et al.^29^ introduced the concept of a rotating jet nozzle, effectively a “composite nozzle”^30^. This nozzle incorporates an impeller, leading to the formation of a high-velocity swirling jet as the fluid exits. The efficacy of this nozzle is intrinsically linked to the design of the impeller and the dimensions of the nozzle structure. Despite the superior theoretical performance of certain uniquely shaped nozzles, their practical application in the oil sector is often hindered by manufacturing and technological limitations.

Regarding the sand-washing efficacy of nozzles, Zhu et al.^31^ focused on the serious problems of mud and sand accumulation in oilfield storage tanks and undertook indoor hydraulic studies using cone-straight type nozzles. Their experimental work delineated the relationships between variables such as discharge volume, depth of sand-washing, size of the scoured pits, and the angle of sand-washing, which were more about trends than exact numerical correlations. From these results, they engineered a sand-washing pipeline network optimized for parameters and found, through field testing, that it could reach a sand-washing coverage rate of up to 99%. In a bid to improve the well-washing efficiency of continuous jetting tools, Khan et al.^32^ and his team designed a porous jet nozzle. They assessed its sand-washing capabilities at various sizes, and through numerical simulations, they assessed the impact of factors like orifice diameter and cone angle on exit pressure and velocity. They discovered that the tool exhibits superior sand-washing performance when the orifice diameter is smaller than 0.75 in. Ortega-Casanova et al.^33^ developed a nozzle with a swirl generator (an impeller), termed a swirler nozzle, to study the impact of swirling jets on sand layers at different distances. Through extensive indoor testing, they observed that when the nozzle-to-sand layer distance equals several tens of times the diameter of the nozzle's outlet and the sand grains are smaller than 1 mm, the diameter and depth of the scour holes created by the swirling jet were about twice those produced by a non-swirling jet. This finding underscored the enhanced sand-washing performance of the swirler nozzle. In response to the challenges of sand sedimentation and wellbore cleaning in horizontal wells, Zhu et al.^34^ introduced a rotating jet based sand removal technique and a corresponding nozzle for sand-washing. They investigated the sand-washing capabilities of this nozzle and the movement behaviors of sand particles through two-phase flow theory and numerical simulations. Their findings indicated that the rotating jet from the nozzle could suspend and reaccelerate sand particles, effectively minimizing secondary sedimentation of sand and thereby improving the washing efficiency of horizontal wells. Qu et al.^35^, focusing on the mechanism of jet sand-washing for well cleaning, developed a rotating jet cleaning apparatus. The core of this device is a rotating nozzle head, which is fitted with several nozzles positioned at the front, side, and rear. The side nozzles' jets are primarily used to drive the head's rotation, thereby generating a swirl field in the annular space of the wellbore, which in turn slows down the sand particle sedimentation. Field applications demonstrated that this tool could finish sand-washing and well cleaning tasks sooner than expected and decrease the necessity for repeated well cleanings. Although numerous studies have been conducted on the sand-washing performance of nozzles, few have taken sand-washing depth as a performance metric for the nozzle. Additionally, almost no studies have holistically considered the correlation of this metric with factors like nozzle design dimensions, physical properties of the fluid and sand particles, and the actual wellbore structure, which results in some shortcomings in these research efforts in improving the sand-flushing efficiency of nozzles.

In summary, although many scholars have conducted research on flushing nozzles’ internal flow field, structural size design and optimization, and flushing performance evaluation, many nozzles with outstanding flushing performance remain theoretical and cannot be applied in practice, due to limitations in practical processing, manufacturing costs, and technology. Despite the fact that a few nozzles have been applied in flushing and well cleaning, there are still some deficiencies in flushing performance analysis and improvement (the main deficiencies are reflected in the structural size optimization and internal flow field analysis on nozzles). In order to improve the efficiency of flushing and well cleaning, reduce the probability of secondary sand deposition and repeated well cleaning, and better increase the production and efficiency of gas wells, this article designs a new jet sand removal device, upon considering the characteristics of strata in the Sichuan-Chongqing region, the wellbore structure of shale gas wells, and specific reasons behind sand production. By comparing it with traditional sand removal devices, the article finds that this device has the dual capabilities of jet flushing and negative-pressure sand-carrying. As the structural size of the flushing nozzle in this device, and physical characteristic parameters of fluid and sand have a significant impact on flushing, numerical simulation and control variable methods are used to simulate the flushing process of this device. Finally, it is found that the length-diameter ratio of the exit cylindrical section of the flushing nozzle, inlet diameter, outlet diameter, and nozzle convergence angle have a significant impact on the flushing performance of the device, and the optimum ranges of these parameters when the device demonstrates the best flushing performance are obtained.

## The whole structure and working principle of the new sand removal device

**Figure 1:**
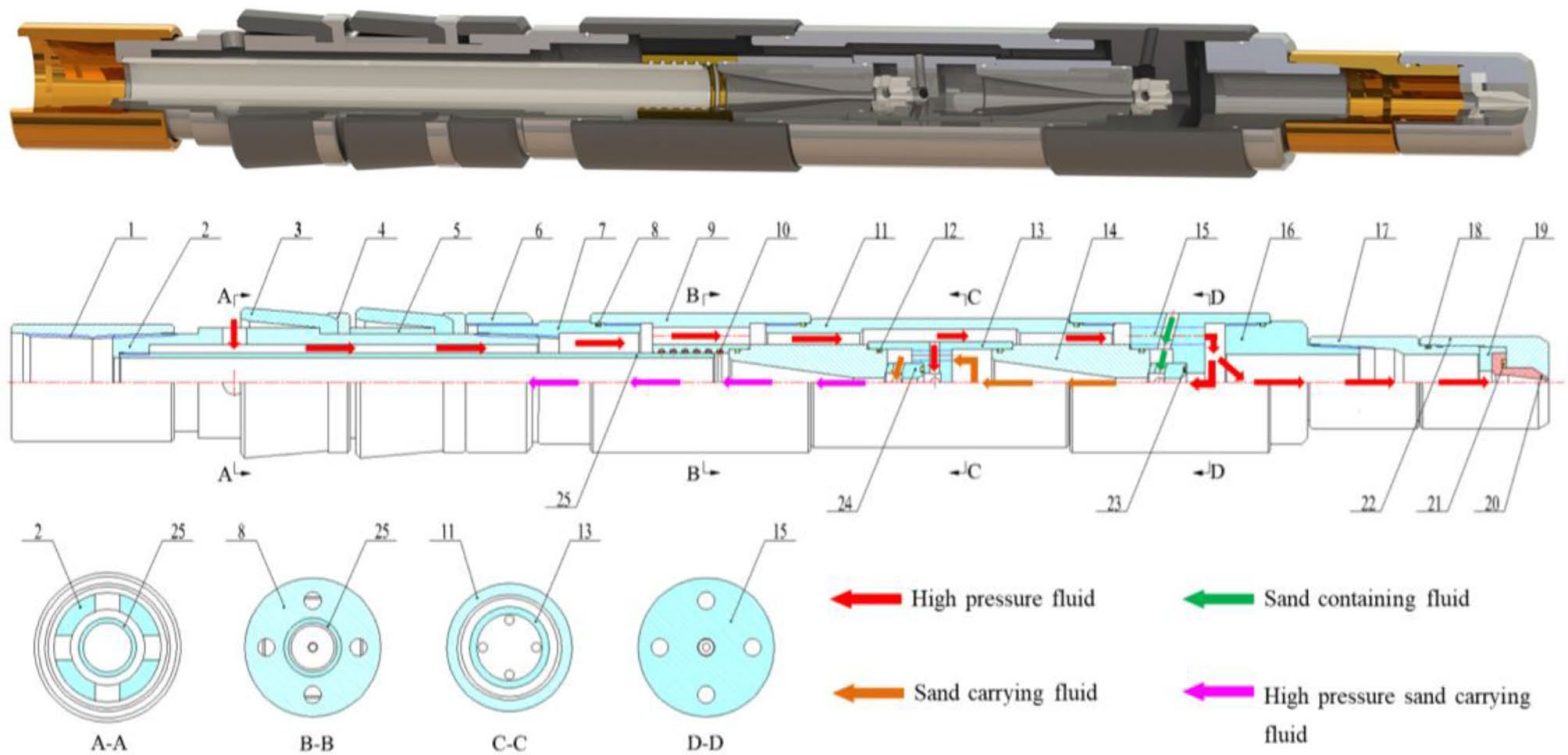
The whole structure and working principle of the novel downhole sand removal device. 1. tubing hanger, 2. central tube, 3. packer cup, 4. support ring, 5. spacer sleeve, 6. adjustable threaded sleeve, 7. lower sub a, 8. seal ring a, 9. upper sub a, 10. seal ring b, 11. connecting tube, 12. seal ring c, 13. upper inlet chamber, 14. diffuser tube, 15. lower inlet chamber, 16. lower sub b, 17. upper sub b, 18. seal ring d, 19. nozzle pressure plate, 20. sand-washing nozzle, 21. seal ring e, 22. nozzle holder, 23. seal ring f, 24. jet nozzle, 25. insert pipe.

The overall structure of the new jet sand-washing device is shown in Fig. 1. The device is assembled from 25 types of components, and the sealing performance of the device is good in various places, with a relatively simple overall structure. Under the action of the booster pump and foam generator located on the ground, the high pressure foam fluid (the flow direction of the high pressure fluid in the device is as shown by the red arrows in Fig. 1) enters the device through the annular space between the tubing and the casing and the small hole in the central tube. The part of the high pressure fluid flows through the internal flow channel of the device, entering into the jet nozzle from the upper inlet chamber, while another part of the high pressure fluid enters into the lower sub b from the lower inlet chamber. Since the lower sub contains a bidirectional flow channel, the fluid is divided into two parts at this point. One part acts as a sand-carrying fluid, which, after being jetted through the sand-washing nozzle, creates an impact on the sand or sand layer at the bottom of the well, causing the settled sand grains to become suspended and to be carried upward along with the high-pressure fluid. Meanwhile, the other part serves as the lift-powered fluid, entering through the nozzle inlet adjacent to the lower inlet chamber and forming a jet at the nozzle outlet. Due to the small internal flow channel of the nozzle, the high pressure fluid creates a suction effect after passing through the nozzle's jet stream. This effect generates a certain negative pressure zone in the vicinity of the nozzle outlet. Consequently, under the action of the pressure differential, the sand-containing fluid (the flow direction of the sand-containing fluid in the device is indicated by the green arrows in Fig. 1) enters the negative pressure zone through the small hole in the lower inlet chamber. Due to the diffuser tube's internal flow channel being a gradually enlarging expansion channel, this expansion channel converts part of the kinetic energy of the sand-carrying fluid (which is formed by the thorough mixing of the sand-containing fluid and the high-pressure fluid) into pressure energy, thereby enhancing the upward return capability of the sand-carrying fluid (the flow direction of the sand-carrying fluid in the device is indicated by the orange arrows in Fig. 1). In order to the sand-carrying fluid's return to the ground more efficiently and to avoid the additional need for a lifting device during the return process, high-pressure foam fluid from the upper inlet chamber is utilized as the power fluid. Once jetted through the nozzle, it generates a suction effect on the pressurized sand-carrying fluid, causing it to mix again with the high-pressure fluid. After passing through the diffuser tube, the pressure of the sand-carrying fluid is further increased. Subsequently, the high-pressure sand-carrying fluid, which has now undergone two stages of pressure enhancement (the flow direction of high-pressure sand-carrying fluid in the device is shown as pink arrows in Fig. 1), flows through components such as the mandrel and tubing. This process enables the high-pressure sand-carrying fluid to return to the ground effectively.

This novel sand-washing device addresses the shortcomings found in existing technologies by utilizing a sand-washing liquid (sand-washing fluid is actually foam fluid, which is described in the following article) that has characteristics such as low density, good stability, minimal leakage, and low damage to the oil reservoir. The device achieves the impact on the sand particles or layers at the bottom of the well (after the high pressure fluid passes through the sand-washing nozzle, the high speed jet can impact the sand settling at the bottom of the well) and carries the sand (using the Venturi ejector principle to lift the sand-containing fluid). Thus, it completes the entire sand removal and well washing process for the sand producing well. After comparing the new sand-washing device with traditional tools (mechanical sand retrieval devices and hydraulic sand-washing devices) (the specific comparison process can be seen in reference^36^), it was found that the former resolves issues such as low sand removal efficiency, difficult well circulation cleaning, and the tendency for sand to resettle that are particularly problematic in wells with severe loss and in long horizontal wells. Due to the device's overall simple structure (lacking moving parts), superior sealing quality (sealing rings installed at all connection points), wide applicability (suitable for use in vertical wells, high-angle wells, and horizontal wells for sand removal), and lower production costs, it also substantially reduces the risk of subterranean safety incidents to a certain extent.

## Comparative analysis and optimization of sand-washing fluids

The sand carrying fluid serves as a medium for transporting sand particles from the well, and the extent of its sand carrying capacity is a key indicator of the fluid's performance quality (Altering the viscosity, density, and specific flow patterns of the sand-carrying fluid will directly affect its performance). Through on-site visits and literature review^37^, we have learned that the sand-carrying fluids commonly used in major domestic oil fields are mainly composed of mixtures such as clear water, mud, and aerated fluids. According to the composition of the media, they can be roughly divided into three categories. The main performance and physical property parameters of these three types of sand carrying media are compared as shown in Table 1. From the Table 1, it can be observed that clear water has the lowest viscosity, moderate density, and the lowest required cost. However, when clear water is used for sand washing in the well, its performance in sand carrying, the damage to the formation, and the subsequent ability to recover the reservoir are all relatively poor. Additionally, major oil fields have rarely used clear water directly as a sand carrying fluid anymore. Nowadays, oil fields are more inclined to use mixtures of clear water with added thickeners or foamed fluids. Compared to clear water, besides requiring higher costs, these alternatives exhibit significantly better sand carrying capacity and cause less damage to the formations. When comparing the two and making an optimal choice, the foam fluid is selected as the working fluid for the new sand-washing device, because of its superior ability to restore the permeability of the reservoir within the well after sand washing, as compared to the mixture of clear water and thickeners.

**Table 1:** Comparison of performance characteristics of different sand-flushing media.

Sand-flushing media	Dynamic viscosity (${10}^{-3}$ Pa s)	Density ($\text{kg}/{\text{m}}^{3}$)	Flow regime category	Friction coefficient	Formation damage	Sand carrying capacity	Reservoir permeability recovery capacity	Required cost
Clear water	1.005	998.2	Newtonian fluid	Large	Severe	Poor	Poor	Low
Foam fluid	4.5–11	500–900	Non-Newtonian fluid	Smaller	Light	Better	Better	Higher
Mixture of clear water and thickening agent	1–10	1000–1500	Non-Newtonian fluid (incomplete)	Smaller	Lighter	Better	Average	Higher

## Establishment of computing methods and simulation models

### Fundamental control equations of fluid motion

During the sand-washing operation in well, once the high-pressure foam fluid proceeds from the casing annulus into the novel sand-washing device, part of this fluid is jetted out through the nozzles within the device. This portion of the fluid becomes the lifting power fluid for the ensuing transportation of sand carrying foam. Meanwhile, another portion of the high pressure foam fluid serves as a sand-washing medium which, after being jetted through the sand-washing nozzle, impacts the sand grains or layers at the well bottom, causing the particles to become suspended and move. It is clear that the sand-washing process represents a multiphase flow that incorporates gas, liquid, and solid phases. As the high-pressure foam fluid contains a relatively low amount of gas and exhibits a significant velocity increase upon ejection from the nozzles, the associated multiphase flow field can be assumed as incompressible turbulent flow. Additionally, the fluid movement is governed by the foundational Navier–Stokes equations that describe fluid dynamics. Therefore, based on the principle of mass conservation and Newton's second law of force, the respective equations are established as follows:

The continuity equation is

1 $$\frac{\partial \rho }{\partial t}+\frac{\partial \left(\rho {u}_{x}\right)}{\partial x}+\frac{\partial \left(\rho {u}_{y}\right)}{\partial y}+\frac{\partial \left(\rho {u}_{z}\right)}{\partial z}=0$$

where $\rho$ is the density of the fluid, kg/${\text{m}}^{3}$; *t* is time, s; and ${u}_{x}{, u}_{y}$ and ${u}_{z}$ are the velocity components of the fluid microelement along the *X*, *Y,* and *Z* axes, m/s.

The momentum equation is

2 $$\left\{\begin{array}{c}\rho \frac{du}{dt}=-\frac{\partial {P}_{0}}{\partial x}+\frac{\partial {\tau }_{xx}}{\partial x}+\frac{\partial {\tau }_{yz}}{\partial y}+\frac{\partial {\tau }_{zx}}{\partial z}+\rho {f}_{x}\\ \rho \frac{dv}{dt}=-\frac{\partial {P}_{0}}{\partial y}+\frac{\partial {\tau }_{yy}}{\partial x}+\frac{\partial {\tau }_{zy}}{\partial y}+\frac{\partial {\tau }_{xy}}{\partial z}+\rho {f}_{y}\\ \rho \frac{dw}{dt}=-\frac{\partial {P}_{0}}{\partial z}+\frac{\partial {\tau }_{zz}}{\partial x}+\frac{\partial {\tau }_{yz}}{\partial y}+\frac{\partial {\tau }_{xz}}{\partial z}+\rho {f}_{z}\end{array}\right.$$

where ${P}_{0}$ is the static pressure, Pa; ${\tau }_{ij}$ is the stress tensor (*i* and *j* can be taken as *x*, *y,* and *z*, respectively), Pa; and ${f}_{k}$ is the volume force of gravity (*k* can be taken as *x*, *y* and *z*, respectively), ${\text{N}/\text{m}}^{3}$.

### Comparison and selection of turbulence models

Considering the turbulent nature of the high pressure foam liquid ejected from the sand-washing nozzle, to enhance the precision of flow field simulations, a suitable turbulence model must be selected. This necessitates a comprehensive comparative analysis, incorporating the design of the sand-washing nozzle, available turbulence models within Computational Fluid Dynamics, and their respective application conditions. In FLUENT software, the prevalent turbulence models can be essentially grouped into four principal categories^38^. These categories include the Spalart–Allmaras (S-A) model, the k-epsilon (k-ε) model, the k-omega (k-ω) model, and the Reynolds Stress Model (RSM). These turbulence model categories can further be subdivided, with the number of equations, advantages and disadvantages, as well as required computational time for each model, as shown in Table 2. Because the entire nozzle sand-washing process encompasses both nozzle jet and boundary layer flows, the Realizable k-epsilon (k-ε) turbulence model was selected for the numerical simulation analysis. This model provides more accurate predictions for dissipation rates in flat plate and cylindrical jet flows and is also well-suited for boundary layer flows, flow separation, and other related phenomena. Hence, the Realizable k-ε model was ultimately chosen for this study. The governing equations of this turbulence model are:

3 $$\frac{\partial }{\partial t}\left(\rho k\right)+\frac{\partial }{\partial {x}_{i}}\left(\rho k{u}_{j}\right)=\frac{\partial }{\partial {x}_{i}}\left[\left(\mu +\frac{{\mu }_{t}}{{\sigma }_{k}}\right)\frac{\partial k}{\partial {x}_{j}}\right]+{G}_{k}+{G}_{b}-\rho \varepsilon -{Y}_{\text{M}}+{S}_{k}$$

4 $$\frac{\partial }{\partial t}\left(\rho \varepsilon \right)+\frac{\partial }{\partial {x}_{j}}\left(\rho \varepsilon {u}_{j}\right)=\frac{\partial }{\partial {x}_{j}}\left[\left(\mu +\frac{{\mu }_{t}}{{\sigma }_{k}}\right)\frac{\partial \varepsilon }{\partial {x}_{j}}\right]+\rho {C}_{\varepsilon }{S}_{\varepsilon }-\rho {C}_{2}\frac{{\varepsilon }^{2}}{k+\sqrt{v\varepsilon }}+{{C}_{1}\frac{\varepsilon }{k}{C}_{3}G}_{b}+{S}_{\varepsilon }$$

where $k$ is the turbulence kinetic energy, ${\text{m}}^{2}/{\text{s}}^{2}$; ${u}_{j}$ is the velocity of the fluid, m/s; $\varepsilon$ is the turbulent dissipation rate, ${\text{m}}^{2}/{\text{s}}^{3}$; *μ* is the dynamic viscosity of turbulence, Pa·s; ${G}_{k}$ is the turbulent kinetic energy caused by the velocity gradient of the laminar flow, ${\text{m}}^{2}/{\text{s}}^{2}$; ${G}_{b}$ is the turbulent kinetic energy caused by buoyancy, ${\text{m}}^{2}/{\text{s}}^{2}$; ${Y}_{\text{M}}$ is the contribution term to the dissipation rate of the turbulent pulsating expansion into the global flow field in the compressible flow; ${C}_{1}$, ${C}_{2}$, and ${C}_{3}$ are three constants; ${\sigma }_{\varepsilon }$ and ${\sigma }_{k}$ are the Prandtl numbers; and ${S}_{k}$ and ${S}_{\varepsilon }$ are user-defined source items.

**Table 2:** Comparative analysis of different turbulence models.

Classification of turbulence models	Name of turbulence model	Number of equations in the model	Advantages	Disadvantages	Computational time
S-A model	S-A model	1	Applicable to wall bounded flow simulation research	Not applicable to uniformly decaying, isotropic turbulent flows	Short
k-ε model	Standard k-ε model	2	Applicable to fully turbulent flow research	Not applicable to near-wall and low Reynolds number transitional layer simulations	Shorter
	RNG k-ε model	2	Applicable to fully turbulent flow, transient flow and applicable to flows with stream curvature	Low Reynolds number flows and flows in near-wall regions still require special treatment	Average
	Realizable k-ε model	2	Applicable to rotational flows, flow separation, jets from flat plates, cylinders, and complex secondary flows	The calculation of rotating and static flow regions does not yield a natural turbulence viscosity	Shorter
k-$\upomega$ model	Standard k-$\upomega$ model	2	Applicable to wall bounded flows and free shear flows	Not applicable to separated flows caused by adverse pressure gradients	Shorter
	SST k-$\upomega$ model	2	Applicable to rotational flow, adverse pressure gradient flow, and transonic shock waves over airfoils	Cannot accurately predict the timing and extent of flow separation from a smooth surface	Shorter
RSM模型	RSM model	7	Applicable to hurricane flow, high-speed rotational flow in combustion chambers, and secondary flow in pipes	The predictive results for axisymmetric jets and the wake behind a disk show significant discrepancies	Longer

### Determination of multiphase flow models

In FLUENT software, there are three commonly used multiphase flow models, which are the Volume of Fluid model (the VOF model), the Mixture model, and the Eulerian model. After researching literature^39^, it was discovered that the VOF model is applicable for computing the flow of immiscible fluids such as air and water. It is frequently used for layered flows, jet breakup processes, the motion of large gas bubbles in liquids, and issues related to dam overflow. The mixture model is a simplified multiphase flow model that is appropriate for computing flow issues where the volume concentration exceeds 10% (such as issues of particle suspension, fluidized beds, etc.). It is commonly used for particle settling processes, cyclone separators, and bubble flows with small volume fractions. Although the Eulerian model is also suitable for computing flow problems with volume concentrations greater than 10%, during the computations, all phases are subject to the same background pressure, and the phases are both distinct from and interactive with each other. During the process of nozzle jet sand-washing (sand layers), due to the volume concentration of sand exceeding 10% (in the simulation model, the nozzle outlet is level with the sand’s surface or 0.2 m away from the topmost surface of the sand layer), and considering that the sand particles will be suspended and transported after being impacted by the fluid, the present study opts to employ the Eulerian model for multiphase flow simulation. Since the formation and collapse of foam is an extremely complex process, and CFD has not yet been able to simulate the flow of foamy fluids, this paper treats the foamy fluid as a single-phase fluid. Furthermore, it defines the fluid parameters in the simulation by measuring the actual physical properties of the foamy fluid, such as its viscosity and density.

### Definition and calculation formula for complete scour depth of the sand layer

After consulting certain literature^40,41^, we have found that many scholars define the performance of sand-washing nozzles by using the outlet velocity of the nozzle or the magnitude of the jet impact force (the calculation formula for the jet impact force also includes the outlet velocity). While both factors significantly influence the sand-washing performance of the nozzle, they are applicable mainly for theoretical and trend analysis. In the actual process, the velocity of the fluid ejected from the nozzle jet experiences reduction and divergence throughout its course. Consequently, by the time the fluid actually contacts the sand, its velocity has substantially decreased. The attenuation and divergence of velocity, along with many other factors, can impact the evaluation of a nozzle’s sand-washing efficiency. To mitigate these influences, the concept of ‘total sand-washing depth has been introduced as a metric for gauging sand-washing performance. The specific formula for total sand-washing depth is:

5 $${L}_{w}={L}_{z}-{L}_{s}$$

where ${L}_{w}$ is the full sand-washing depth, m; ${\text{L}}_{\text{z}}$ is the furthest sand-washing distance, m; ${\text{L}}_{\text{s}}$ is the maximum depth of the sand pit after sand-washing completion, m.

### The selection of nozzle structure for the sand-washing nozzle

**Figure 2:**
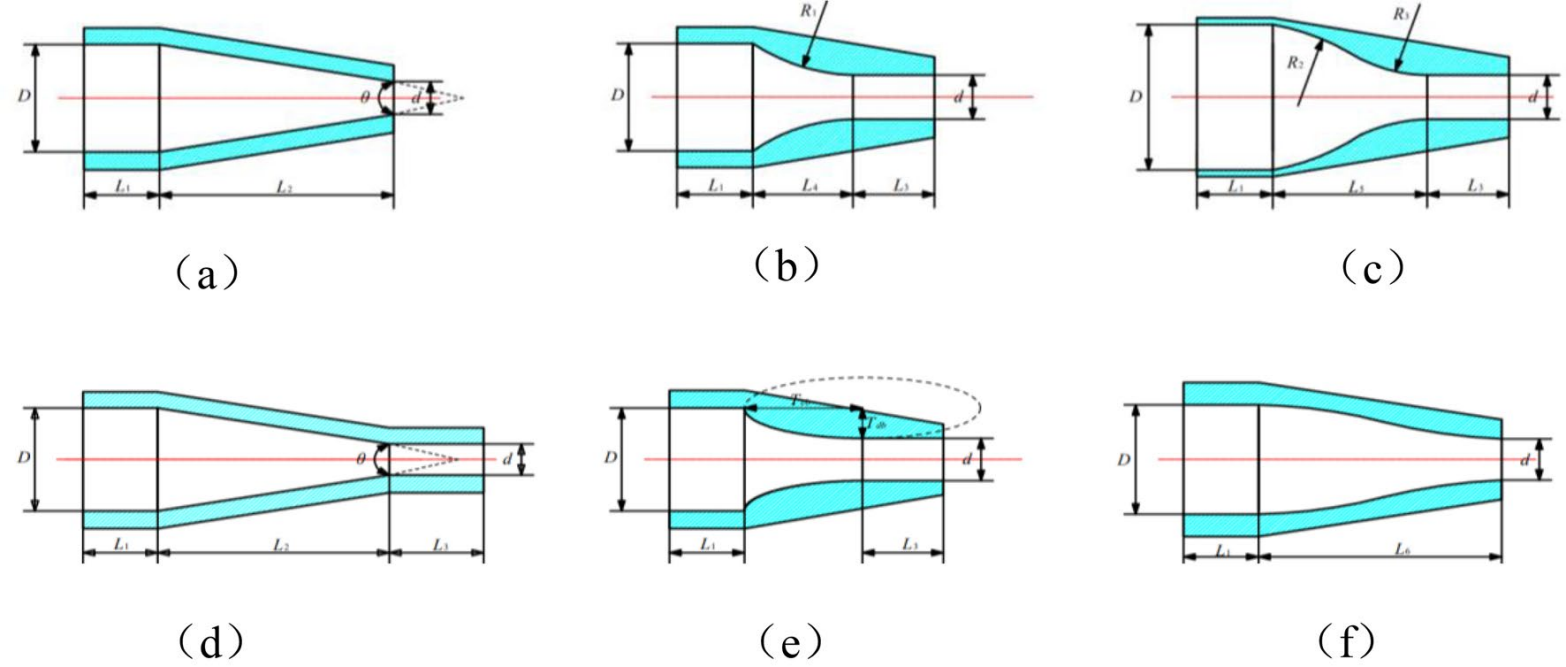
Two-dimensional structural diagrams of several typical nozzles where $\text{D}$ is the nozzle inlet diameter, $\text{mm}$; $\text{d}$ is the nozzle outlet diameter, $\text{mm}$; $\uptheta$ is nozzle contraction angle, °; ${\text{L}}_{1}$ is nozzle inlet cylindrical section length, mm; ${\text{L}}_{2}$ is nozzle contraction section length, mm; ${\text{L}}_{3}$ is nozzle outlet cylindrical section length, mm; ${\text{L}}_{4}$ is contraction section length of single arc nozzle, mm; ${\text{L}}_{5}$ is contraction section length of double arc nozzle, mm; ${\text{L}}_{6}$ is constriction section length of streamlined nozzle, mm; ${R}_{1}$ is the arc radius of single arc nozzle, mm; ${\text{R}}_{2}$ is radius of the first arc segment in the double arc nozzle, mm;${\text{R}}_{3}$ is radius of the first arc second in the double arc nozzle, mm; ${\text{T}}_{\text{cb}}$ is major axis radius of the elliptical nozzle, mm; ${T}_{db}$ is minor axis radius of the elliptical nozzle, mm. (**a**) Cone nozzle, (**b**) single arc nozzle, (**c**) double arc nozzle, (**d**) cone straight nozzle, (**e**) elliptical nozzle, (**f**) streamlined nozzle.

The novel jet sand-washing device comprises three nozzles (two jet nozzles and one sand-washing nozzle). When the high pressure foam fluid jets from the device’s sand-washing nozzle, it generates an impact on the sand or sand bed at the bottom of the well, suspending the sedimented sand. The jet nozzles employ the Venturi jet entrainment principle to twice increase the pressure of the sand carrying foam fluid. It is evident that the device achieves efficient sand removal from the wellbore through a combination of jet impact on the sand layer and negative pressure sand carrying flow. To enhance the sand removal efficiency of the device, it is foremost to increase the jetting impact capability of the sand-washing nozzle. Only by converting as much sedimented sand into a suspended state as possible, it can be feasible to maximize the clearance of more sedimented sand in the well. To increase the sand removal efficiency of the device, it is foremost necessary to enhance the jetting impact capacity of the sand-washing nozzle. By consulting certain literature^39^, we have identified six typical nozzles. Figure 2 shows the specific structures of the various nozzles, from which we can observe that the internal flow paths of nozzles (b), (c), (e), and (f) are of special shapes. Although the jetting performance of these nozzles with unique shapes is superior, they are not yet widely used in the petroleum industry due to limitations in actual manufacturing processes and technological constraints. When taking into consideration factors such as production costs and safety, the field tends to favor the selection of either (a) or (d). To enhance the cohesiveness of the jet, increase the depth of sand-washing, and align with actual field conditions, this paper selects the cone straight nozzle (d) as the sand-washing nozzle for the novel sand-washing device.

### Establishment of simulation model and setting of boundary conditions

**Figure 3:**
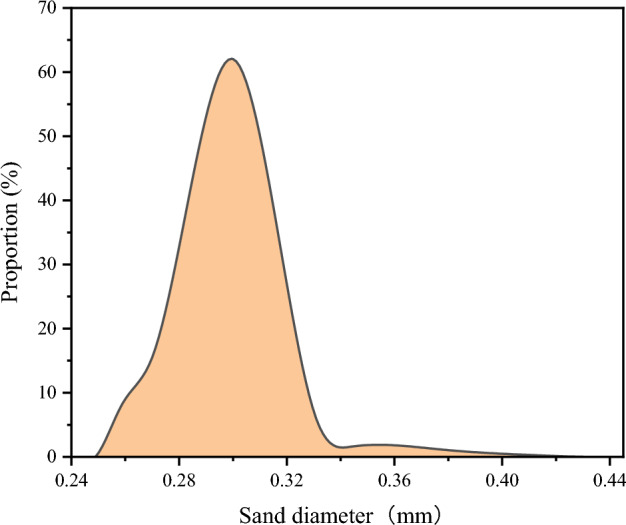
Sand particle size distribution.

**Figure 4:**
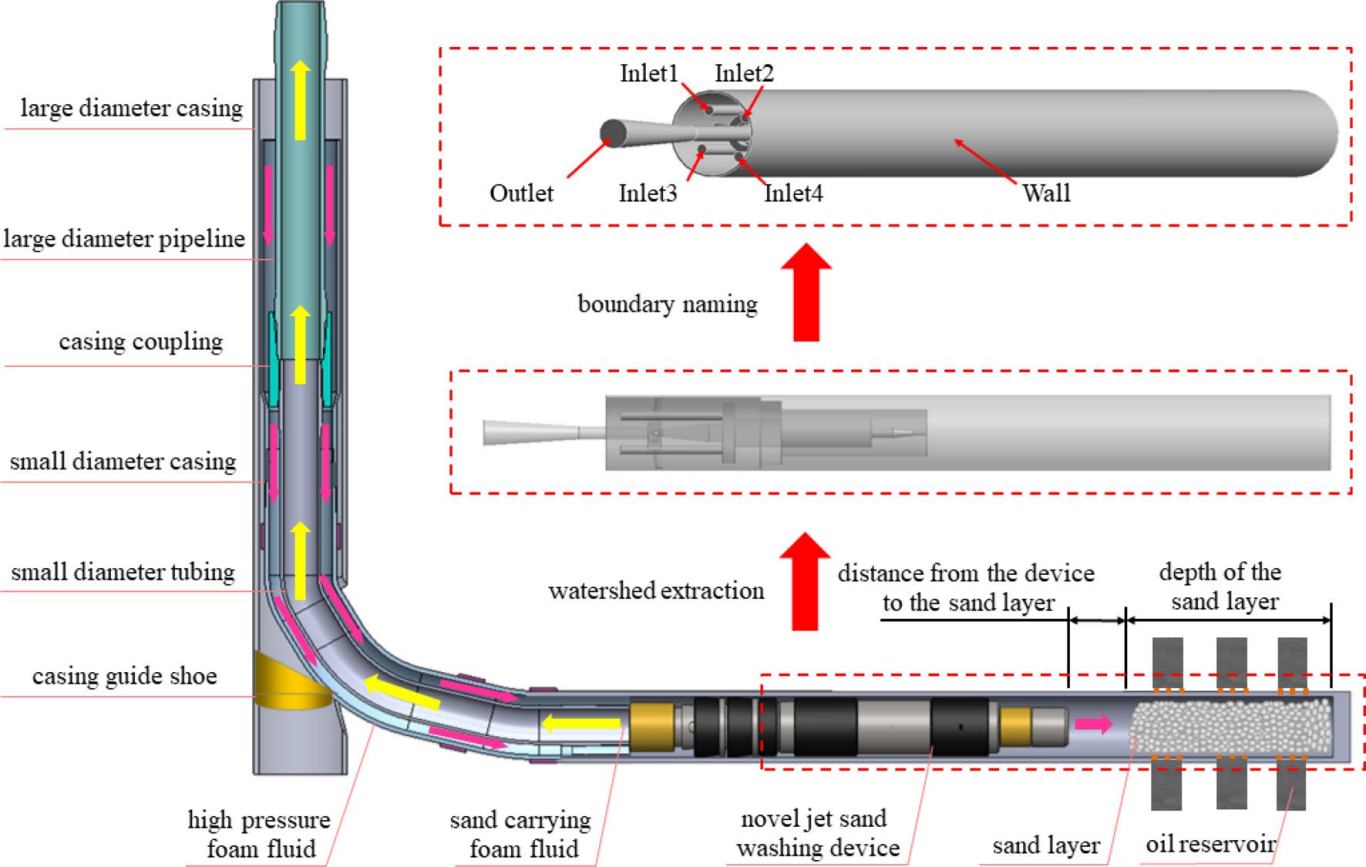
Sand-washing simulation model of novel sand removal device.

In order to establish a numerical simulation model for the novel sand-washing device, a simulation model of the device's sand-washing process is developed based on key structural dimensions of the device, physical parameters of the foam fluid and sand particles (the actual size of sand is around 50 mesh, As shown in Fig. 3, the proportion of sand particles with a diameter of 0.3 mm is the highest), sand layer depth, and wellbore size, among others (specific values for these parameters can be found in Table 3). Due to the complex internal structure of the novel sand-washing device, both the 3D modeling software SOLIDWORKS (2016 version, URL link: http://www.solidworks.com/zh-hans) and the numerical simulation software ANSYS FLUENT (2020 version, URL link: http://www.ansys.com/zh-cn/products/fluids/ansys-fluent) are employed to construct the sand-washing simulation model. The overall structure of this simulation model is illustrated in Fig. 4, from which it can be observed that a total of three types of boundaries have been set on the simulation model: four inlet boundaries (Inlet1, Inlet2, Inlet3, Inlet4), one outlet boundary (Outlet), and one wall boundary (Wall). The wall boundary specifically refers to all remaining surfaces within the simulation model that have not been defined. Based on the actual working conditions on site, the total flow rate of the novel sand-washing device has been determined to be 6.33 L/s. Since there are four inlets in the simulation model, each with a diameter of 14 mm, the fluid velocity at each inlet is calculated to be approximately 10.28 m/s after unit conversion. The outlet boundary in the model is set as a pressure outlet with a pressure value of 6 MPa.

**Table 3:** The specific values of main parameters in the simulation model.

Main parameter names	Units	Numerical values
Distance from the sand-washing nozzle to sand layer	m	0.2
Sand layer depth	m	1
Casing inner diameter	mm	118
Inlet diameter of the sand-washing nozzle	mm	24
Length of the cylindrical section at the inlet of the sand-washing nozzle	mm	12
Angle of the sand-washing nozzle	°	14
Outlet diameter of the sand-washing nozzle	mm	8
Length of the cylindrical section at the outlet of the sand-washing nozzle	mm	16
Inlet diameter of the jet nozzle	mm	30
Length of the cylindrical section at the inlet of the jet nozzle	mm	20
Angle of the jet nozzle	°	14
Outlet diameter of the jet nozzle	mm	8
Length of the cylindrical section at the outlet of the jet nozzle	mm	3.3
Diameter of the throat	mm	19.5
Length of the throat	mm	25.5
Distance from the throat to the nozzle	mm	13
Angle of the diffusion tube	°	8
Length of the diffusion tube	mm	163.8
Density of sand grains	kg/${\text{m}}^{3}$	2600
Diameter of sand grains	mm	0.3
Density of foam fluid	kg/${\text{m}}^{3}$	600
Viscosity of foam fluid	mPa s	40

## Mesh generation and independence verification for the simulation model

In the process of meshing for simulation models, to lower the computational time and enhance the precision of the numerical simulation outcomes, the implications of varying mesh sizes and configurations on the simulation results are analyzed by altering the mesh dimensions and structure. This approach is undertaken to identify the optimal mesh size and shape. From Table 4, it is evident that three distinct grid geometries and five different grid sizes were selected, resulting in a total of fifteen meshing methods for the simulation model. Furthermore, expansion layers were incorporated at predetermined boundaries such as the inlet and outlet. Then, each model with completed grid division was analyzed individually through numerical simulation.

**Table 4:** Grid independence verification of simulation model.

Meshing method	Grid shape	Minimum meshing size (mm)	Maximum meshing size (mm)	Number of grid nodes	Total number of grid faces	Total number of units	Maximum velocity at the nozzle outlet (m/s)
#1	Tetrahedron	1	20	135,547	859,544	370,968	74.43
#2		2	20	114,991	717,106	307,682	74.76
#3		3	20	76,877	470,317	200,328	75.07
#4		4	20	52,937	318,769	135,009	75.34
#5		5	20	40,775	243,562	102,830	75.72
#6	Hybrid of tetrahedron and hexahedron	1	20	160,763	1,080,947	474,287	74.11
#7		2	20	121,583	777,231	335,856	74.14
#8		3	20	79,592	494,493	211,514	74.12
#9		4	20	55,160	340,257	145,194	74.55
#10		5	20	43,034	264,291	112,498	74.91
#11	Polyhedron	1	20	431,178	688,497	145,229	74.11
#12		2	20	359,461	579,026	123,174	74.12
#13		3	20	239,618	392,007	84,371	74.45
#14		4	20	165,850	274,153	59,646	74.78
#15		5	20	128,850	217,504	48,307	75.04

After comparing the simulation results of these partitioning methods, it was found that when the simulation model is divided by using a tetrahedral mesh, as the minimum mesh size is gradually reduced, the maximum velocity at the outlet of the sand-washing nozzle also decreases, but this velocity value is still not stable (this indicates that the accuracy of the results obtained by dividing the model with a tetrahedral mesh is not high. If one wishes to improve the precision of the simulation results, it will be necessary to continue to reduce the minimum size of the tetrahedral mesh). When using a mixed mesh of tetrahedrons and hexahedrons to partition the simulation model, it is noticeably observed that the maximum velocity at the outlet of the sand-washing nozzle hardly changes when the minimum mesh size is 3 mm or smaller (it is evident that the minimum mesh size for the mixed grid should be 3 mm, which is identified as mesh partitioning method #8 from Table 4). Meanwhile, when employing a polyhedral mesh to divide the simulation model, it becomes apparent that the maximum velocity at the outlet of the sand-washing nozzle no longer varies when the minimum mesh size reaches 2 mm or smaller (it is evident that the appropriate minimum size for the polyhedral grid should be 2 mm, as indicated by mesh partitioning method #12 in Table 4). Since this velocity value is the same as the maximum velocity at the outlet of the sand-washing nozzle when stabilized after simulation using the mixed grid of tetrahedrons and hexahedrons (the velocities are both 74.12 m/s), and the maximum velocity values at the outlet of the sand-washing nozzle, as derived from several classification methods in this table, fluctuate between 74.11 and 75.72 m/s. This indicates that the simulation results not only validate the grid independence of the model well but also further demonstrate that the simulation results have good convergence. In order to reduce the computation time of the finite element simulation, the further comparison was made between the #8 and #12 grid division method. Due to the total number of elements resulting from the #8 grid division methods was 1.72 times that of the #12 grid division method, the finite element simulation computational time for the former is longer than that of the latter. Consequently, the decision was made to adopt the #12 grid division method for the discretization of the simulation model in the subsequent analysis.

## Verification of simulation results accuracy

**Figure 5:**
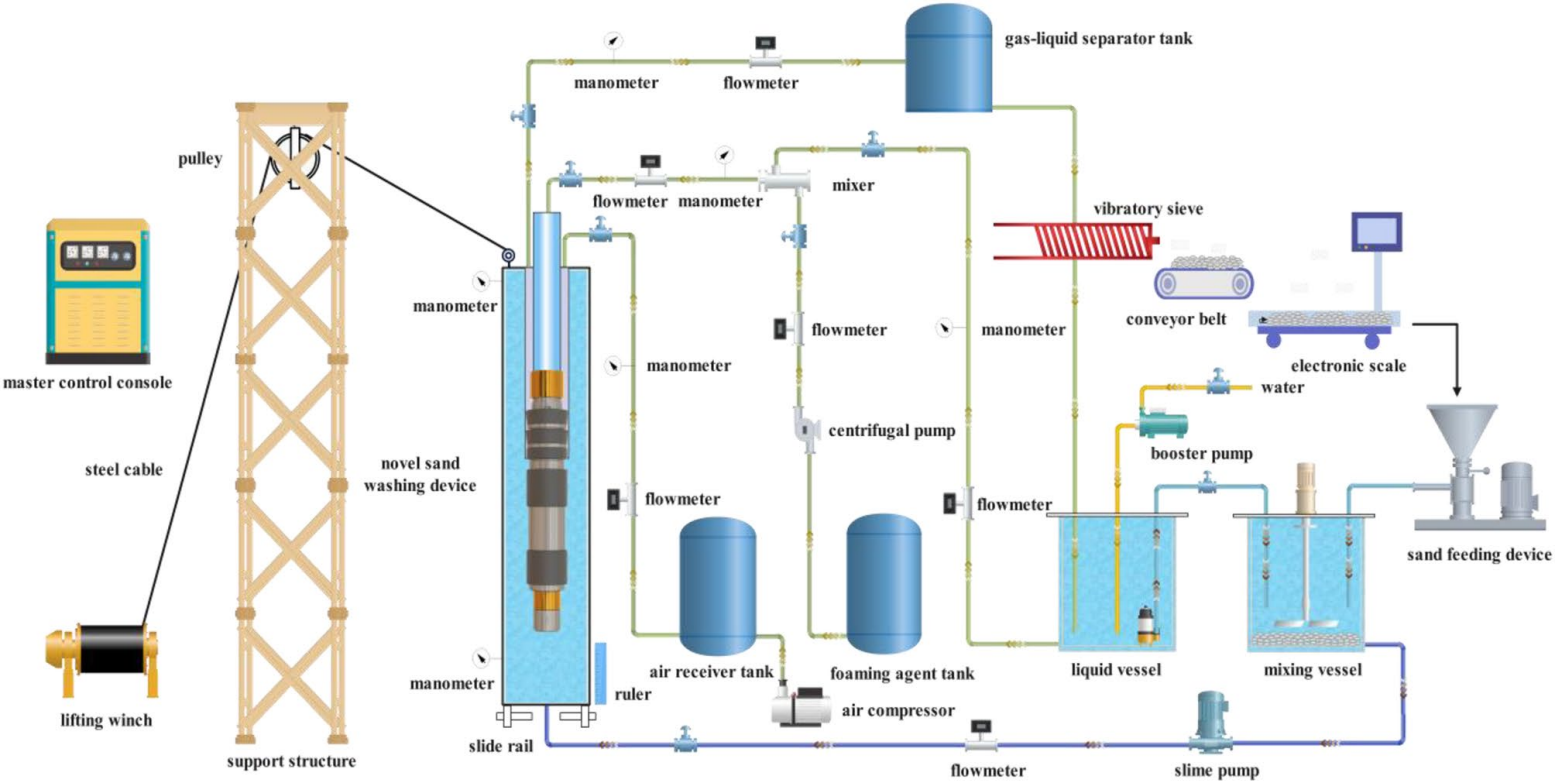
Schematic diagram of the overall structure of the multiphase flow experimental platform.

To verify the accuracy of the simulation results, the established multiphase flow experimental platform (This platform comprises components such as a solid–liquid-gas three-phase separation, mixing, and recirculation system, a solid-phase particle injection and collection system, as well as a data acquisition and control system) is utilized to carry out indoor experiments on the sand-washing process of the novel downhole sand-washing device. The overall structure of this multiphase flow experimental platform is shown in Fig. 5. The platform is capable of simulating the sand carrying and sand-washing processes in vertical wells of 30 m in length, highly deviated wells, and horizontal wells (The inclination angle of the simulated wellbore is adjusted by using devices like winches and pulleys). Furthermore, the fluid used for sand carrying and washing within this platform can be either a single-phase liquid (such as clear water) or a gas–liquid two-phase fluid (such as foamy liquid). To observe the movement of sand in the wellbore, the entire wellbore is constructed using transparent glass tubing, and high-speed cameras are employed to capture the motion of the sand in the wellbore. The platform additionally incorporates an automatic sand feeding system, granular sand weighing apparatus, and facilities for initial blending of sand grains with designated fluids. Moreover, the sand-bearing fluid that is circulated out of the wellbore is subjected to solid, liquid, and gas phase separation and subsequent collection. By drying and weighing the collected sand the remaining mass of sand in the wellbore can be determined, thus evaluating the sand-carrying capacity of a certain fluid or the sand-washing capability of a particular device.

The analysis of the sand washing process for a novel downhole sand washing device in a vertical simulated wellbore was carried out using the experimental platform (the experimental details and procedures are elaborately described in reference^36^). In the experiment, the sand layer thickness was artificially set to 1.8 m (by pre-filling the simulated wellbore with 0.3 mm sand and gravel, and using a ruler to measure the thickness of the sand layer to meet the experimental requirements). The installation position of the device was 0.2 m from the upper surface of the sand layer, and the position of the device was kept constant throughout the entire sand washing process. After the experiment concludes, it is necessary to dry, weigh, and record data pertaining to the sand particles carried out by the foam solution from the wellbore. Ultimately, the recorded experimental data should be compared with the results obtained from numerical simulation. As illustrated in Fig. 5, after a detailed analysis of the simulation results and the data obtained from the experiments (the total mass of sand carried out by the foam fluid), it is found that when the sand washing time is constant in the simulation results, the total mass of sand extracted from the wellbore steadily decreases as the outlet diameter of the sand washing nozzle increases. Conversely, with a constant nozzle outlet diameter, the total mass of sand retrieved from the wellbore gradually increases with the increase of sand washing time. However, after 10 s, the mass of the sand remains almost unchanged. At this point, the total mass can be approximately defined as the overall sand washing capacity of the new downhole sand washing device equipped with that particular nozzle. After the total mass of sand extracted from the wellbore ceases to vary significantly, a one-to-one comparison between the specific simulation results and the corresponding experimental data reveals that the simulation results closely approximate the experimental outcomes (Following certain calculations, it is discovered that the error values for these three sets are all less than 10%), thereby validating the correctness of the simulation model presented in this paper and the accuracy of the simulation results.

After conducting an analysis on the simulation results curve for the total mass of sand carried out from the wellbore over time, it was found that the entire process of the change in total mass of sand with time can be defined in four stages. These four stages are specifically:

### Initial sand discharge stage (Region I in Fig. 6)

**Figure 6:**
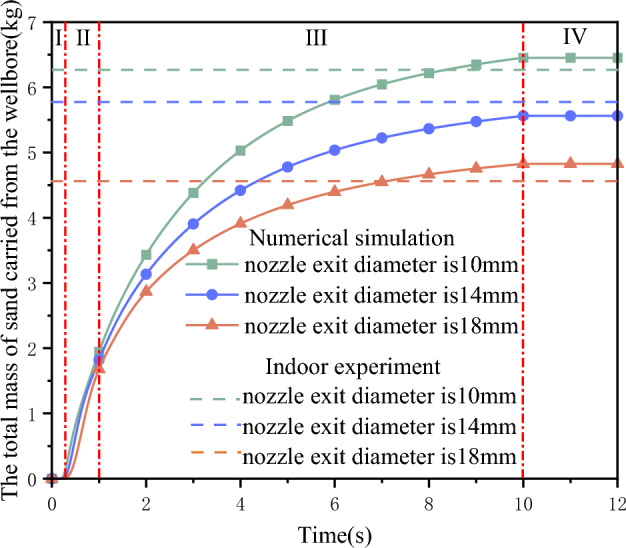
Comparison between experimental results and simulation results.

This stage specifically refers to the initiation of impact on the bottom-hole sand particles or sand layer by a high-pressure foam fluid jetting from the sandblasting nozzle, which causes some sand at the well bottom to become suspended, and are eventually carried up with the high-pressure foam fluid. Considering the wellbore has a certain length and the initial velocity of sand particles is 0, during an extremely short period after the commencement of sand washing, no sand particles have been expelled from the wellbore yet. As a result, throughout this brief interval, the total mass of sand particles brought out from the wellbore remains at 0.

### Linear sand discharge stage (Region II in Fig. 6)

This stage specifically refers to the commencement of expulsion of the high-pressure foam fluid with sand from the wellbore, and with the passage of time, the total mass of sand being transported out of the wellbore gradually increases. As the novel downhole sand removal device is situated relatively close to the upper surface of the sand layer, during the initiation of sand washing, a substantial quantity of sand becomes suspended and can be carried up out of the wellbore along with the high-pressure foam fluid. Resulting in a trend where the total mass of sand carried out from the wellbore exhibits a linear variation with time.

### Sand transitional discharge stage (Region III in Fig. 6)

This stage specifically refers to the commencement of expulsion of the high-pressure foam fluid continues to carry sand from the wellbore, and with the passage of time, the total mass of sand being transported out of the wellbore gradually decreases. The reason is that the sand washing capacity of the novel downhole sand removal device decreases as the depth of the well washing completed increases. Therefore, during this time period, although the total mass of sand carried out from the well continues to increase, the curve representing the total mass of sand over time shows a continuously decreasing slope.

### Sand mass stabilization stage (Region IV in Fig. 6)

This stage specifically refers to the condition where the high-pressure foam fluid can only carry a minimal amount of sand, or is no longer able to transport sand out of the wellbore. The reason is that the maximum sand washing depth of the novel downhole sand removal device has approached or become less than the current distance between the device and the upper surface of the sand layer. Consequently, during this time period, almost no sand particles can be expelled from the wellbore, resulting in the total mass of sand particles carried out from the wellbore essentially remaining unchanged.

## Analysis of the effect factors of nozzle structure on sand washing depth

### Effect of outlet cylindrical section length-to-diameter ratio on sand washing depth

The outlet cylindrical section length-to-diameter ratio refers to the value obtained by dividing the length of the straight pipe section (the transition section) at the nozzle outlet by the outlet diameter in a cone straight nozzle. This dimensionless parameter has a direct impact on factors such as the velocity at the nozzle outlet and indirectly influences the quality of the nozzle jet performance. It is evident that the outlet cylindrical section length-to-diameter ratio is one of the significant factors affecting the sand-washing performance of cone straight nozzle.

**Figure 7:**
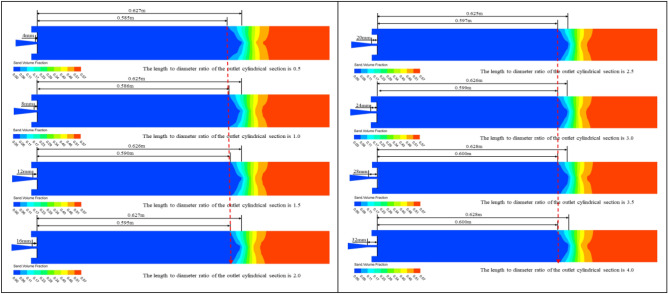
Cloud diagrams of sand particle volume fraction during the sand washing process with nozzles of different outlet cylindrical section length-to-diameter ratios.

In order to investigate the effect of the length-to-diameter ratio of the outlet cylindrical section on sand washing depth, the length of the outlet straight pipe section was altered while keeping all other conditions constant. This allowed for the analysis of the relationship between different outlet straight pipe section lengths (different outlet cylindrical section length-to-diameter ratios) and the sand washing performance of the nozzle. As shown in Fig. 7, when the length of the outlet straight pipe section is 4 mm (the outlet cylindrical section length-to-diameter ratio is 0.5), the full sand washing depth is 0.585 m, while the farthest sand washing distance is 0.627 m. It can be clearly observed that as the length of the outlet straight pipe section increases, the farthest sand washing depth remains almost unchanged, while the full sand washing depth increases, the rate of increase in the the full sand washing depth is diminishing. When the length of the outlet straight pipe section is greater than or equal to 24 mm (the outlet cylindrical section length-to-diameter ratio is 3.0), the depth of the sand pit no longer decreases significantly.

**Figure 8:**
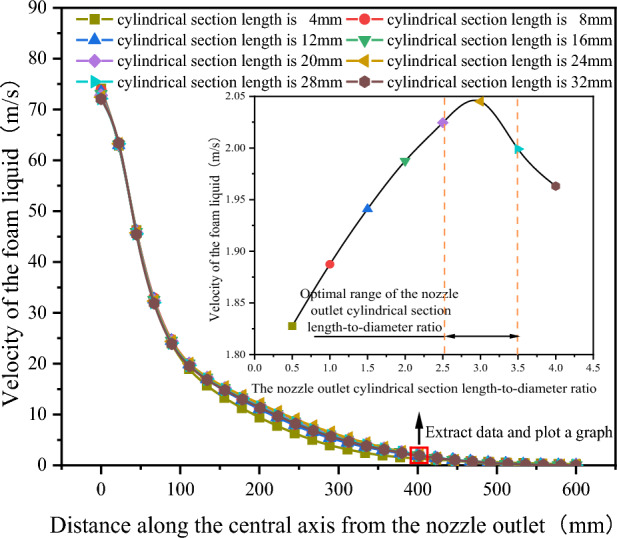
Velocity variation curve of foam fluid jetted from the nozzle along the central axis.

As shown in Fig. 8, after extracting the velocity variation curves of the foam fluid jetted from the nozzles (with different outlet cylindrical section length-to-diameter ratios) along the central axis, It was found that as the distance from the nozzle outlet increases along the central axis, the velocity of the foam fluid ejected from the nozzle gradually decreases. Interestingly, the magnitude of the velocity change initially increases before decreasing. Additionally, the trend of these velocity changes is not affected by variations in the length-to-diameter ratio of the outlet cylindrical section. To compare the jet performance of nozzles with different outlet cylindrical section length-to-diameter ratios, the velocity of the foam fluid at a distance of 400 mm along the central axis from the nozzle outlet was measured (this position is located within the fundamental section of the submerged jet theory, where the fluid flow is stable), and the data were plotted in a curve graph. After analyzing the curve graph, it was observed that as the length of the cylindrical section at the nozzle outlet increases (with the increase of outlet cylindrical section length-to-diameter ratio), the velocity of the foam fluid first rises and then decreases, and the length of the cylindrical section at the nozzle outlet is 24 mm, the foam fluid has the highest speed (jet velocity decay is the slowest), this infers that the nozzle jet performance is optimized when the outlet cylindrical section length-to-diameter ratio is 3. However, the actual optimal length-to-diameter ratio of the outlet cylindrical section might not be this exact value, it is clearly evident that the optimum range for the length-to-diameter ratio of the nozzle outlet cylindrical section between 2 and 4.

### Effect of nozzle outlet diameter on sand washing depth

During the design process of nozzle structures, the primary dimensional parameter that the primary dimensional parameter that typically needs to be determined is the nozzle outlet diameter, and the selection of this diameter should also consider the practical application of the nozzle. If the nozzle outlet diameter is too large, the jetting performance of the nozzle will be significantly diminished. On the other hand, if the nozzle outlet diameter is too small, there is an increased likelihood of the nozzle outlet will become blocked during processes such as sand washing in wells, oil sludge removal, and perforation.

**Figure 9:**
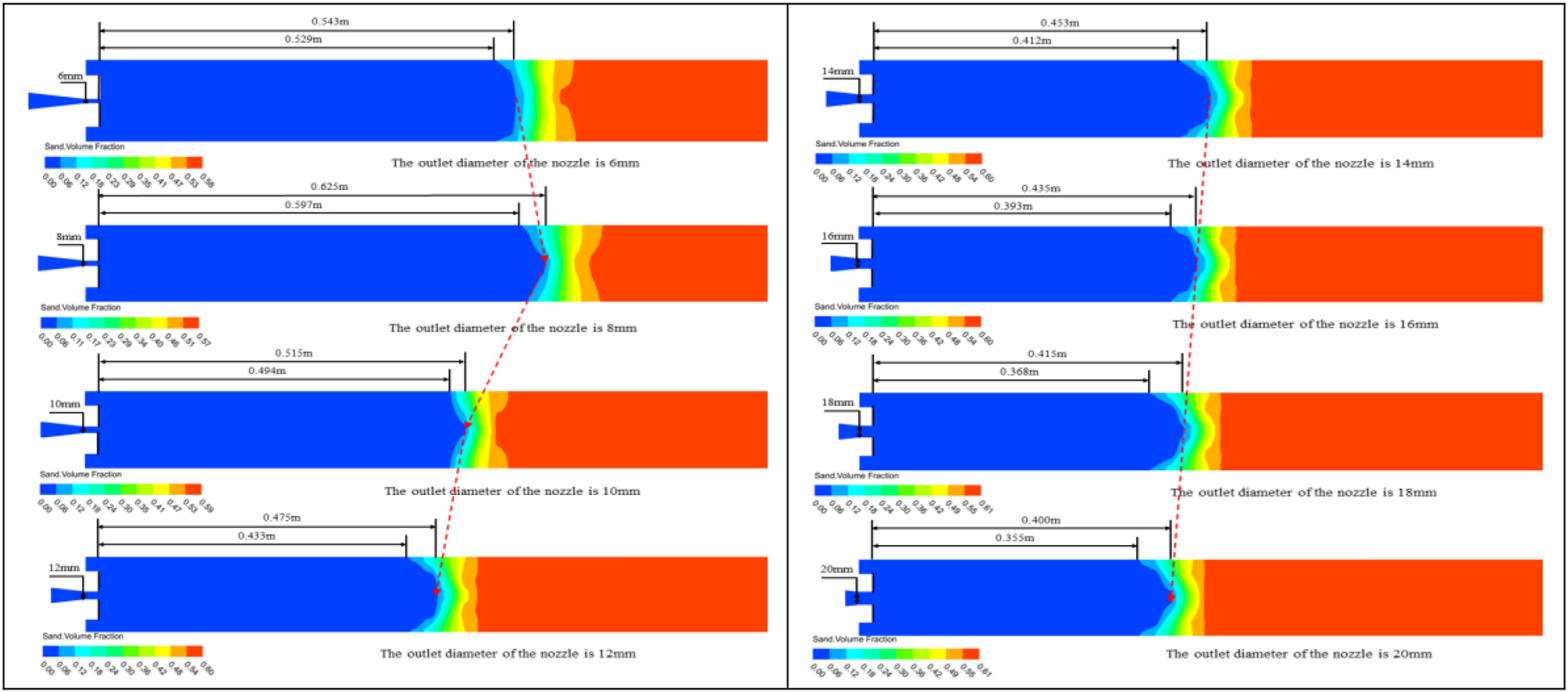
Cloud diagrams of sand particle volume fraction during the sand washing process with nozzles of different outlet diameters.

In order to investigate the effect of the nozzle outlet diameter on sand washing depth, the nozzle outlet diameter was altered while keeping all other conditions constant. This allowed for the analysis of the relationship between different nozzle outlet diameters and the sand washing performance of the nozzle. As shown in Fig. 9, when the nozzle outlet diameter is 6 mm, the full sand washing depth is 0.529 m, while the farthest sand washing depth is 0.543 m. It can be clearly observed that both the full sand washing depth and the furthest sand washing depth are increasing (when the outlet diameter changes from 6 to 8 mm). Once the nozzle outlet diameter exceeds 8 mm, it was found that both the furthest sand-washing depth and the full sand washing depth begin to decrease, and the rate of decrease is quite significant.

**Figure 10:**
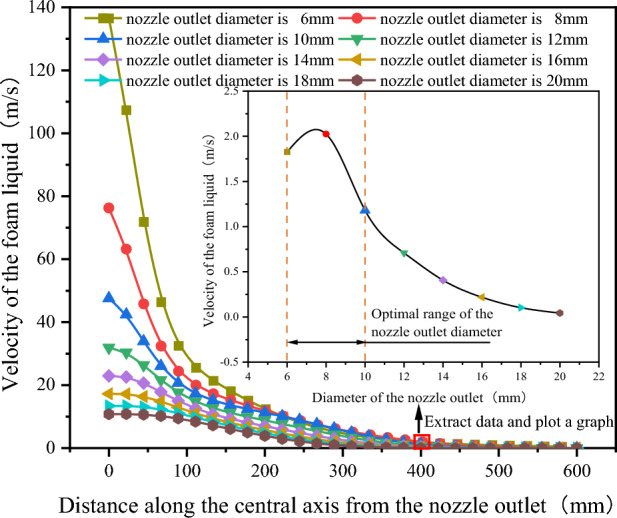
Velocity variation curve of foam fluid jetted from the nozzle along the central axis.

As shown in Fig. 10, after extracting the velocity variation curves of the foam fluid jetted from the nozzles (with different nozzle outlet diameters) along the central axis, It was found that as the distance from the nozzle outlet increases along the central axis, the velocity of the foam fluid jetted from the nozzle and the amplitude of its velocity variations both decrease progressively. Moreover, the trend of the foam fluid velocity change is influenced by variations in the nozzle outlet diameter. To compare the jet performance of nozzles with different outlet diameters, the velocity of the foam fluid at a distance of 400 mm along the central axis from the nozzle outlet was measured, and the data were plotted in a curve graph. After analyzing the curve graph, it was observed that as the nozzle outlet diameter increases, the velocity of the foam fluid first rises and then decreases, and the nozzle outlet diameter is 8 mm, the foam fluid has the highest speed, this infers that the nozzle jet performance is optimized when the nozzle outlet diameter is 8 mm. However, the actual optimal the nozzle outlet diameter might not be this exact value, it is clearly evident that the optimum range for the nozzle outlet diameter between 6 and 10 mm.

### Effect of nozzle contraction angle on sand washing depth

The nozzle contraction angle is one of the primary parameters influencing fluid jetting. The selection of its specific numerical value typically involves two main considerations: firstly, the amount of resistance encountered by the fluid as it passes through the contraction angle, and secondly, whether the width of the fluid stream compromises the jetting efficiency of the nozzle. Generally speaking, if the nozzle contraction angle is excessively large, the fluid will experience significant frictional resistance while passing through the constriction angle. Conversely, if the nozzle contraction angle is too small, the fluid stream may narrow due to the Coanda effect, thereby impairing the fluid’s jetting impingement capability.

**Figure 11:**
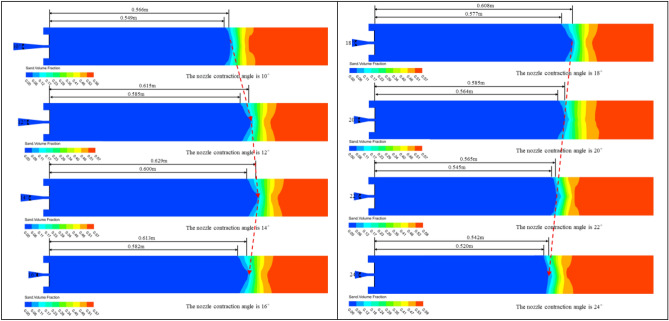
Cloud diagrams of sand particle volume fraction during the sand washing process with nozzles of different nozzle contraction angles.

In order to investigate the effect of nozzle contraction angle on sand washing depth, the nozzle contraction angle was altered while keeping all other conditions constant. This allowed for the analysis of the relationship between different nozzle contraction angles and the sand washing performance of the nozzle. As shown in Fig. 11, when the nozzle contraction angle is 10°, the full sand washing depth is 0.549 m, while the farthest sand washing depth is 0.566 m. It can be clearly observed that both the full sand washing depth, the furthest sand washing depth, and the depth of the sand pit are increasing (when nozzle contraction angle changes from 10° to 14°). Once the nozzle contraction angle exceeds 14°, it was found that both the furthest sand-washing depth and the full sand washing depth begin to decrease, and the rate of decrease is quite significant.

**Figure 12:**
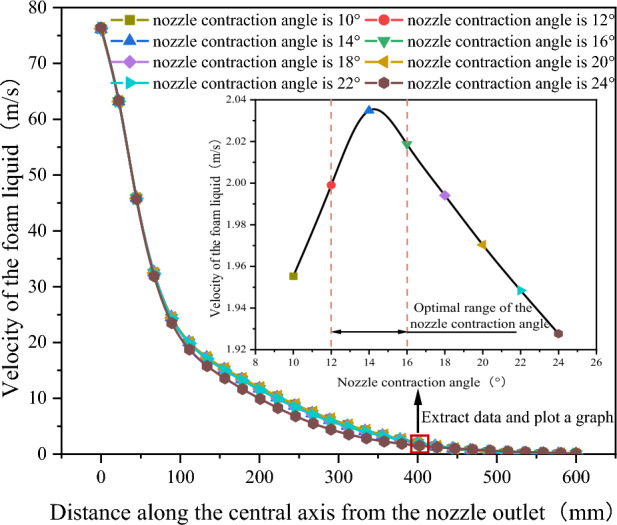
Velocity variation curve of foam fluid jetted from the nozzle along the central axis.

As shown in Fig. 12, after extracting the velocity variation curves of the foam fluid jetted from the nozzles (with different nozzle contraction angles) along the central axis, It was found that as the distance from the nozzle outlet increases along the central axis, the velocity of the foam fluid jetted from the nozzle and the amplitude of its velocity variations both decrease progressively. Moreover, the trend of the foam fluid velocity change is not influenced by variations in the nozzle contraction angle. To compare the jet performance of nozzles with different outlet diameters, the velocity of the foam fluid at a distance of 400 mm along the central axis from the nozzle outlet was measured, and the data were plotted in a curve graph. After analyzing the curve graph, it was observed that as the nozzle contraction angle increases, the velocity of the foam fluid first rises and then decreases, and the nozzle contraction angle is 14°, the foam fluid has the highest speed, this infers that the nozzle jet performance is optimized when the nozzle contraction angle is 14°. However, the actual optimal the nozzle contraction angle might not be this exact value, it is clearly evident that the optimum range for the nozzle contraction angle between 12° and 16°.

### Effect of nozzle inlet diameter on sand washing depth

The nozzle inlet diameter is one of the key parameters in the design of the nozzle structure, has a definite impact on the performance of the nozzle jet depending on its specific size. Under the condition that the nozzle contraction angle remains constant, changing the size of the nozzle inlet diameter will cause the contraction section of the nozzle to change. With the nozzle inlet diameter remaining constant, altering only the size of the contraction angle will also induce changes in the contraction section of the nozzle. It is evident that changing either the nozzle inlet diameter or the nozzle contraction angle will result in similar effects.

**Figure 13:**
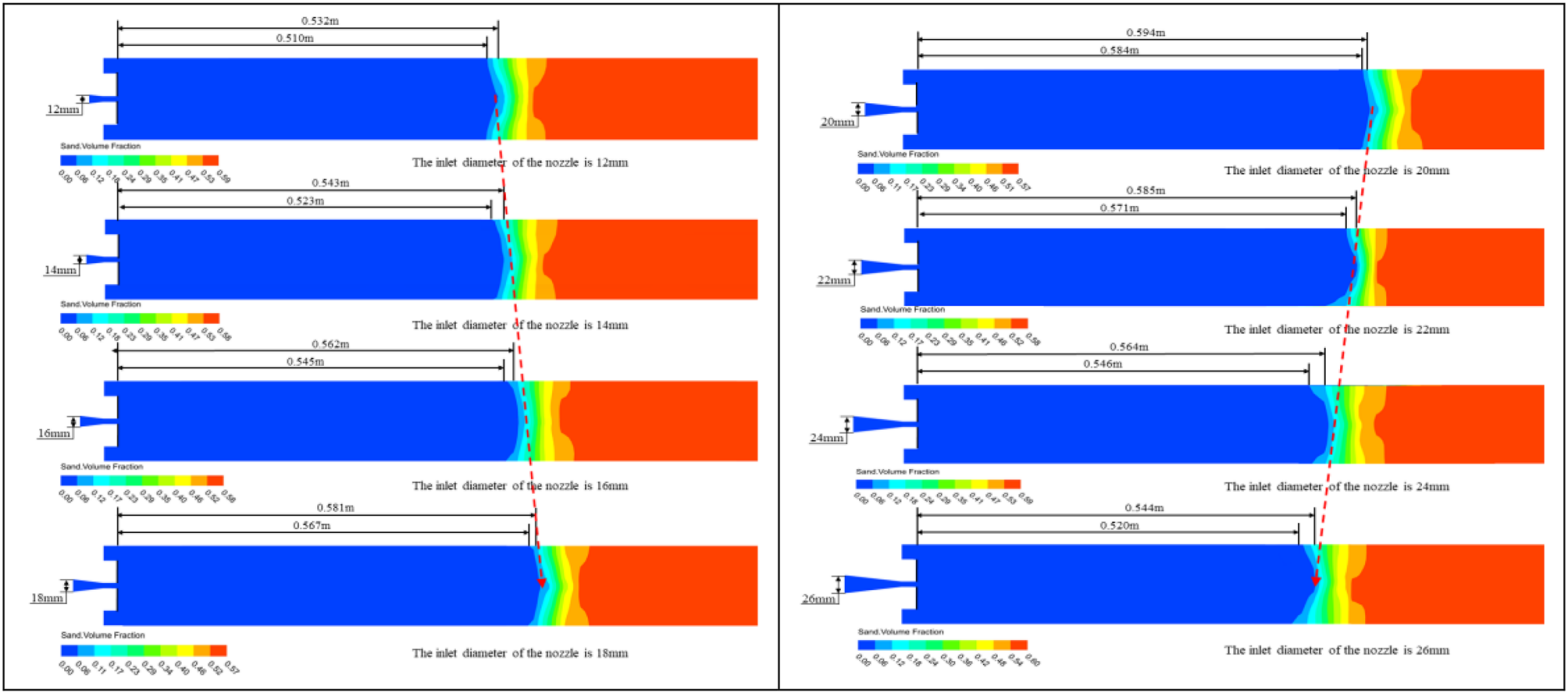
Cloud diagrams of sand particle volume fraction during the sand washing process with nozzles of different nozzle inlet diameters.

In order to investigate the effect of nozzle inlet diameter on sand washing depth, the nozzle inlet diameter was altered while keeping all other conditions constant. This allowed for the analysis of the relationship between different the nozzle inlet diameter and the sand washing performance of the nozzle. As shown in Fig. 13, when the nozzle inlet diameter is 12 mm, the full sand washing depth is 0.519 m, while the farthest sand washing depth is 0.535 m. It can be clearly observed that both the full sand washing depth and the furthest sand washing depth are increasing (when the nozzle inlet diameter changes from 12 to 20 mm), but the depth of the sand pit is decreasing. Once the nozzle inlet diameter exceeds 20 mm, it was found that both the furthest sand-washing depth and the full sand washing depth begin to decrease, and the rate of decrease is quite significant.

**Figure 14:**
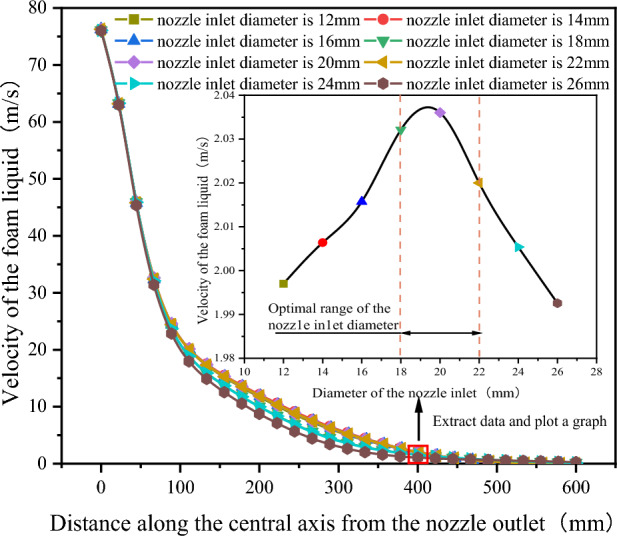
Velocity variation curve of foam fluid jetted from the nozzle along the central axis.

As shown in Fig. 14, after extracting the velocity variation curves of the foam fluid jetted from the nozzles (with different nozzle inlet diameters) along the central axis, It was found that as the distance from the nozzle outlet increases along the central axis, the velocity of the foam fluid jetted from the nozzle and the amplitude of its velocity variations both decrease progressively. Moreover, the trend of the foam fluid velocity change is not influenced by variations in the nozzle inlet diameter. To compare the jet performance of nozzles with different inlet diameters, the velocity of the foam fluid at a distance of 400 mm along the central axis from the nozzle outlet was measured, and the data were plotted in a curve graph.

After analyzing the curve graph, it was observed that as the nozzle inlet diameter increases, the velocity of the foam fluid first rises and then decreases, and the nozzle inlet diameter is 20 mm, the foam fluid has the highest speed, this infers that the nozzle jet performance is optimized when the nozzle inlet diameter is 20 mm. However, the actual optimal the nozzle contraction angle might not be this exact value, it is clearly evident that the optimum range for the nozzle inlet diameter between 18 and 22 mm.

## Conclusion

1. To bolster the gas extraction efficiency of the shale gas wells in the middle and late stages in the Sichuan-Chongqing region, mitigate the risk of sand burying the production layer, and achieve cost reduction and efficiency improvement, a new jet sand removal device has been devised. Designed to address the shortcomings of existing technology, the device utilizes the characteristics of foam liquid, such as low density, good stability, low leakage, and low damage to the oil layer, to blast and convey sand particles or sand layers at the bottom of the wells, thus completing the entire process of sand removal and well flushing in wells producing sand. Through a comparison of the device with traditional sand removal tools, it is uncovered the device boasts a simple overall structure, excellent sealing performance, wide applicability, and lower required production costs. Additionally, it can tackle the issues of low sand removal efficiency and challenges in circulating well flushing in complex wells, including severe absorption wells and long horizontal wells.
2. To enhance the sand-washing and unblocking performance of the new jet sand removal device, various sand-washing fluids and the structures of different sand-washing nozzles are compared for selection. Taking into account production costs, sand-washing and conveying capabilities, formation damage, and reservoir recovery capabilities, foam fluid is chosen as the sand-washing fluid for the device. Considering the limitations of actual processing and technology, manufacturing costs, jet compactness, and safety, a conical nozzle is selected as the sand-washing nozzle for the device. To some extent, these measures have beefed up the device’s overall sand-washing and unblocking performance.
3. By utilizing indoor experimental data as a control group, the accuracy of the simulation model and results for sand-washing and unblocking in the new jet sand removal device is validated. Upon analyzing the simulation curve depicting the change of the total mass of sand particles carried out of the well shaft over time, it is observed that the entire variation of sand particle mass with time can be categorized into four stages: the initial upward movement stage of sand particles, the linear discharge stage of sand particles, the transitional discharge stage of sand particles, and the stable stage of sand particle quality.
4. After delving into the factors impacting the sand-washing and unblocking performance of the new jet sand removal device one by one through numerical simulation and control variable method, it is discovered that the length diameter ratio of the cylindrical segment of the nozzle outlet, the outlet diameter, and the contraction angle of the nozzle substantially affect the device's sand-washing performance. Additionally, according to the numerical simulation results, to attain the best sand-washing performance, the optimum ranges for the length-diameter ratio of the cylindrical segment of the nozzle outlet, the outlet diameter, the contraction angle of the nozzle, and the inlet diameter are 2 to 4, 6 mm to 10 mm, 12° to 16°, and 18 mm and 22 mm, respectively.

## Data Availability

The datasets used and/or analyzed during the current study are available from the corresponding author upon reasonable request.

## References

[1] Zheng X (2022). Progress and prospects of oil and gas production engineering technology in China. Pet. Explor. Dev..

[2] Aguilera RF, Ripple RD, Aguilera R (2014). Link between endowments, economics and environment in conventional and unconventional gas reservoirs. Fuel.

[3] Zou C (2018). Theory, technology and prospects of conventional and unconventional natural gas. Pet. Explor. Dev..

[4] Zhang D (2022). Development prospect of natural gas industry in the Sichuan basin in the next decade. Nat. Gas Ind. B.

[5] Wang ZL (2021). Study on cuttings carrying principle and numerical simulation analysis of new drill pipe. Chin. J. Eng. Des..

[6] Hu D, Xu S (2013). Opportunity, challenges and policy choices for China on the development of shale gas. Energy Policy.

[7] Song Y, Ranjith PG, Wu B (2020). Development and experimental validation of a computational fluid dynamics-discrete element method sand production model. J. Nat. Gas Sci. Eng..

[8] Wan R (2011). Advanced Well Completion Engineering.

[9] Song Y (2024). A comprehensive study of fines migration in internally unstable natural gas hydrate reservoirs. Powder Technol..

[10] Wang S (2018). Shale gas exploitation: Status, problems and prospect. Nat. Gas Ind. B.

[11] Sun Y, Du B, Zhou X (2006). Research on mechanical sand-bailing technology. Acta Pet. Sin..

[12] Ye Z, Zhao Y, Pang Y, Hu Y, Jiang Q (2023). Mechanisms and experimental research on sand transport and settlement of a new sand cleaning system. Arab. J. Sci. Eng..

[13] Qu H (2013). Research on hydraulic parameters and field tests of horizontal wells sand-flushing with rotating jets. Energy Sources A Recov. Util. Environ. Eff..

[14] Li G, Huang Z, Tian S, Shen Z (2010). Research and application of water jet technology in well completion and stimulation in China. Pet. Sci..

[15] Pan Y, Zhai S, Meng X, Pei K, Huo F (2022). Study on the fracturing of rock by high-speed water jet impact. Processes.

[16] Yang L, Li Y, Wang D, Guo Y, Li D (2022). Research on deposition particles carrying with washing tools during well cleaning. J. Pet. Sci. Eng..

[17] Li, G. *Research on concentric tube jetting and negative pressure sand washing technology in horizontal well. Master's Thesis* (China University of Petroleum Huadong, 2016).

[18] Urazmetov O, Cadet M, Teutsch R, Antonyuk S (2021). Investigation of the flow phenomena in high-pressure water jet nozzles. Chem. Eng. Res. Des..

[19] Kim WH, Park TS (2013). Effects of noncircular inlet on the flow structures in turbulent jets. J. Appl. Math. Phys..

[20] Jiang T, Huang Z, Li J, Zhou Y, Xiong C (2022). Experimental investigation of internal and external flow fields of jetting nozzles with different structures. J. Pet. Sci. Eng..

[21] Horisawa H, Sawada F, Onodera K, Funaki I (2008). Numerical simulation of micro-nozzle and micro-nozzle-array flowfield characteristics. Vacuum.

[22] Jiang TW, Huang ZW, Li JB, Zhou YS (2021). Internal flow mechanism of cone-straight nozzle. Pet. Sci..

[23] Jiang TW, Huang ZW, Li JB (2022). Large-eddy simulation of flow characteristics near the wall of cone-straight nozzle. Pet. Sci. Bull..

[24] Chen J, Guo L, Hu Y, Chen Y (2018). Internal structure of a jet nozzle for coalbed methane mining based on airfoil curves. Shock Vib..

[25] Ouyang, M. *Optimal design and experiments study on hydraulic sandblasting perforation nozzle. Master's Thesis* (Chongqing University, 2014).

[26] Zhang X, Li X, Nie S, Wang L, Dong J (2021). Study on velocity and pressure characteristics of self-excited oscillating nozzle. J. Braz. Soc. Mech. Sci. Eng..

[27] Zhou Z, Ge Z, Lu Y, Zhang X (2017). Experimental study on characteristics of self-excited oscillation pulsed water jet. J. Vibroengineering.

[28] Yang S, Tan F, Zhang Y, Liu Z, Jin S (2010). Numerical simulation on internal flow field in cavitating nozzle. Sci. Technol. Eng..

[29] Liao HL, Li GS, Niu J-L, Huang ZW (2013). Integrating-straight & swirling jets bit design and its rock breaking characteristics for radial horizontal hole drilling. J. China Coal Soc..

[30] Li G, Zhang X, Huang Z (2011). Experimental study on rock breaking by a combined round straight jet with a swirling jet nozzle. At. Sprays.

[31] Zhu, H. & Ren, Z. Z. Experimental study on hydraulic sand washing performance of sand washing nozzle. *China Pet. Mach.* **12,** 13–15+26+83, (2005).

[32] Khan JA, Irawan S, Padmanabhan E, Al-Kayiem HH, Rai S (2020). Optimization of coiled tubing nozzle for sand removal from wellbore. J. Pet. Explor. Prod. Technol..

[33] Ortega-Casanova J, Campos N, Fernandez-Feria R (2011). Experimental study on sand bed excavation by impinging swirling jets. J. Hydraul. Res..

[34] Zhu XH, Li JN, Tong H (2013). Mechanism analysis and process optimization of sand and plug removal with rotating jet in horizontal well. J. Cent. South Univ..

[35] Qu H (2011). Hydraulic parameters of sand-flushing with rotating jets in horizontal wells. Pet. Drill. Tech..

[36] Wang, Z. L. *Research on the Characteristics of Sand Particle Movement Within the Wellbore of Sand Producing Oil Wells and Negative Pressure Jet Sand Washing Technology. Doctoral's Thesis* (Yangtze University, 2024).

[37] Li J, Misselbrook J, Sach M (2010). Sand cleanouts with coiled tubing: Choice of process, tools and fluids. J. Can. Pet. Technol..

[38] Rodriguez S (2019). Applied Computational Fluid Dynamics and Turbulence Modeling: Practical Tools, Tips and Techniques.

[39] Sultan RA, Rahman MA, Rushd S, Zendehboudi S, Kelessidis VC (2019). Validation of CFD model of multiphase flow through pipeline and annular geometries. Part. Sci. Technol..

[40] Wen J, Qi Z, Behbahani SS, Pei X, Iseley T (2019). Research on the structures and hydraulic performances of the typical direct jet nozzles for water jet technology. J. Braz. Soc. Mech. Sci. Eng..

[41] Xu K (2020). CFD-based study of nozzle section geometry effects on the performance of an annular multi-nozzle jet pump. Processes.

